# An uncommon case of an infected ovarian endometrioma

**DOI:** 10.1016/j.ijscr.2024.110204

**Published:** 2024-08-22

**Authors:** Abderrahim Siati, Meryem El Jawhari, Mohamed Dehayni

**Affiliations:** Department of Gynecology and Obstetrics, Sheikh Khalifa Ibn Zaid International University Hospital, Mohammed VI University of Sciences and Health, Casablanca, Morocco

**Keywords:** Ovarian endometrioma, Infection, Adnexectomy

## Abstract

**Introduction and importance:**

Ovarian endometriosis is common, most often associated with other endometriotic lesions of the pelvis. Among the classic complications of endometriosis, secondary infection is described but rare. We report a very interesting case of an endometrioma cyst mimicking a tubo ovarian abscess. We describe our diagnosis and surgical management by laparoscopic approach.

**Case presentation:**

A case of a 32-year-old patient, with a history of surgery for ovarian and deep peritoneal endometriosis, who consulted urgently for pelvic pain syndrome in a febrile context. Emergency laparoscopic surgery showed secondary infection of the endometriosic cyst with the presence of pus. A right adnexectomy was performed. The postoperative follow-up was simple.

**Discussion:**

Secondary infection of endometriomas is a classically described but rare complication. Severe endometriosis would be a risk factor for developing a tubo-ovarian abscess with an incidence of 2.3 % in patients with endometrioma. Laparoscopic endometrioma surgery has demonstrated its superiority beyond question, numerous trials have confirmed these data. The benefit is established in terms of pain, infectious risk, scarring, wall complications, adhesion risk, length of hospitalization, return to normal activity, thromboembolic risk and cost.

**Conclusion:**

Infection of an endometriosic cyst is an exceptional complication. It is necessary to emphasize the particularities of this surgery, namely careful dissection to avoid secondary lesions, particularly digestive.

## Highlights

• Secondary infection of endometriomas is a classically described but rare complication. Severe endometriosis would be a risk factor for developing a tubo-ovarian abscess with an incidence of 2.3% in patients with endometrioma.
• We report a case of a 32-year-old patient, with a history of surgery for ovarian and deep peritoneal endometriosis, who consulted urgently for pelvic pain syndrome in a febrile context.
• In this work, we present an exceptional case of an infected ovarian endometrioma which simulates an ovarian tubo abscess. We describe our diagnosis and surgical management by laparoscopic approach.

## 1 Introduction

Endometriosis affects 10 % of women of childbearing age [1]; among them, the proportion of ovarian involvement varies around 40 to 50 % [2,3]. Compared to peritoneal endometriosis, ovarian endometriosis is less often painful [4] and very rarely isolated (1 % of cases in certain series) [5]. Patients with only ovarian endometriosis describe dysmenorrhea in 77 % of cases, intermenstrual pain in 62 % of cases and dyspareunia in 39 % of cases. Infection of endometriosis lesions is rare. Adhering to the SCARE 2023 guidelines, we report a case of an infected ovarian endometrioma with surgical management [6].

## 2 Patient and observation

### 2.1 Patient information

32-year-old patient, single, with a history of ovarian and deep peritoneal endometriosis, underwent surgery three years ago with removal of a right endometriotic cyst. The evolution was marked by the appearance of a new right ovarian endometrioma which measures approximately 77 mm, but the patient only complains of dysmenorrhea and some intermenstrual pain. Subsequently, she benefited from hormonal and symptomatic treatment with regular monitoring every three months.

### 2.2 Clinical findings

Clinical examination shows a fever of 40° and diffuse abdominal contracture suggesting peritonitis.

### 2.3 Diagnostic assessment

**Fig. 1:**
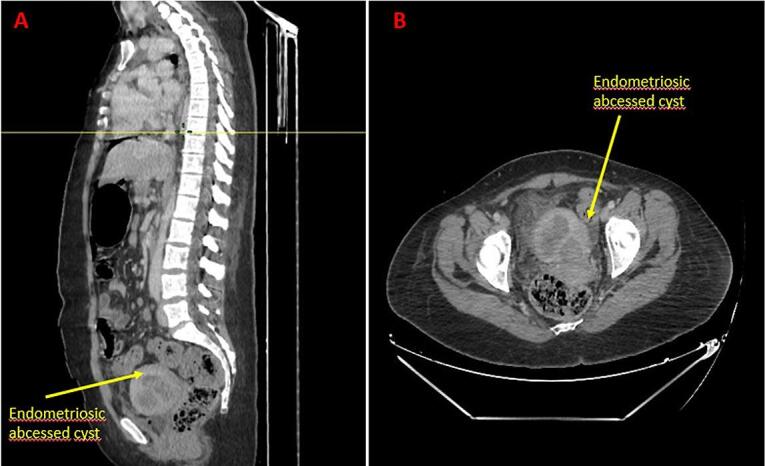
Abdominal pelvic scan showing an ovarian endometrioma giving the appearance of an ovarian tubo abscess.

The abdominal and pelvic scan revealed a right lateral uterine mass giving the appearance of a tubal ovarian abscess and an impure effusion of medium abundance (Fig. 1). The biological assessment showed an inflammatory syndrome with: hyper leukocytosis, elevated C-reactive protein and procalcitonin.

### 2.4 Therapeutic intervention

**Fig. 2:**
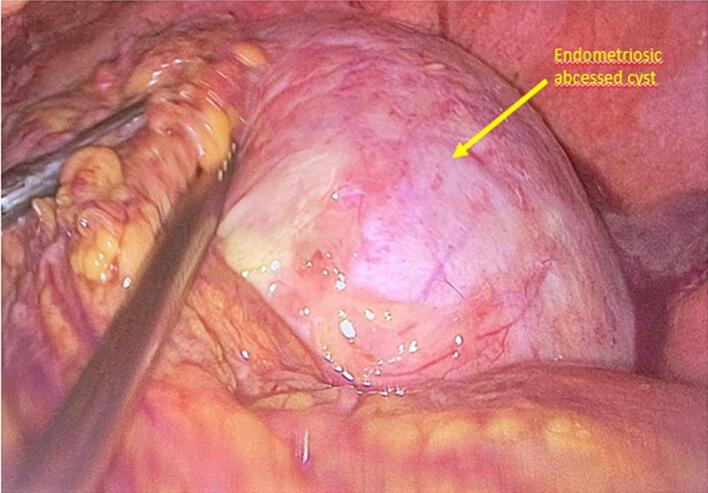
Laparoscopic image showing an infected ovarian endometrioma.

After conditioning, the patient was admitted to the operating room. A laparoscopic surgery was performed, having revealed: an infected ovarian endometrioma with the presence of pus, multiple digestive and omental adhesions and impure blood effusion (Fig. 2). Furthermore, we note the presence of deep endometriosis nodules, and the appendix was of normal size and appearance. We decided to perform a right adnexectomy which was difficult in the presence of digestive adhesions. Firstly: Bacteriological and cytological samples were taken. A careful adhesiolysis was carried out using: dissection forceps, the suction cannula and thermofusion energy (Ligasure®). Secondly: the contents of the “chocolate liquid” cyst were aspirated into the endobag, then we exteriorized the entire right ovary and fallopian tube. Abundant washing with physiological saline was carried out with placement of a redon drain.

### 2.5 Follow-up

Subsequently, the patient was admitted to the intensive care unit for three days, broad-spectrum antibiotic therapy was started. Bacteriological samples and pathology results revealed an endometriotic cyst infected with *Escherichia coli*. The postoperative course was simple; the patient was discharged on the fifth postoperative day. Treatment with LH-RH analogues was initiated.

**Fig. 3:**
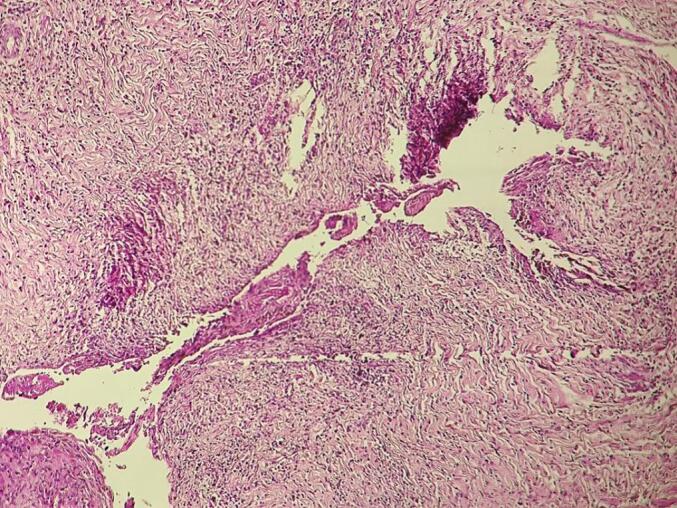
Morphological appearance showing on standard HE staining an endometriotic cyst largely remodeled by a polymorphic inflammatory infiltrate, with the presence of a fibrino-leukocyte coating on the surface.

The pathological examination revealed an endometriotic cyst largely remodeled by a polymorphic inflammatory infiltrate, with the presence of a fibrino-leukocyte coating on the surface (Fig. 3).

## 3 Discussion

Secondary infection of endometriomas is a very rare complication. Severe endometriosis would be a risk factor for developing a tubo-ovarian abscess with an incidence of 2.3 % in patients with endometrioma [7,8]. It occurs most frequently as a complication of egg retrieval in the context of medically assisted procreation [9], but can also occur de novo [10]. The pathophysiology is poorly understood. Infection of endometrioma could be secondary to an alteration of the immune environment within the endometrial glands and stroma. The accumulation of blood during cycles in a cystic space, and the weakness of the wall of the endometriotic cyst compared to a normal ovarian epithelium could promote bacterial proliferation. The usual semiology of endometrioma, in clear T1 hypersignal greater than the fat, persisting on the sequences with saturation of the fat signal associated with a characteristic “shading” in T2, is therefore modified. In the present case, the mass was already known as endometrioma and had the classic features of a tubo-ovarian abscess on CT.

Laparoscopic endometrioma surgery has demonstrated its superiority beyond question [11], numerous trials have confirmed these data. The benefit is established in terms of pain, infectious risk, scarring, wall complications, adhesion risk, length of hospitalization, return to normal activity, thromboembolic risk and cost. The technique of choice is intraperitoneal cystectomy. Whatever the theory used in the formation of endometrioma: there is a starting point for endometrioma at the level of the ovarian cortex which is located in 90 % of cases at the level of the ovary-subovarian dimple adhesion. The cyst therefore does not need to be incised on its anti-mesial edge, adhesiolysis of the cyst in fact leads to its rupture and the release of “chocolate” liquid (this is probably toxic for the peritoneum peritoneal and intracystic washing will be carried out). This adhesiolysis is carried out using scissors and a suction cannula by performing a rocking movement along the pelvic wall, from back to front. The movement begins at the posterior part of the ovary adhering to the subovarian fossa and is continued anteriorly towards the utero-ovarian. Annexectomy may be justified in the event of multiple recurrences in a patient who has previously had a correctly performed cystectomy or in a patient no longer wishing to become pregnant, particularly after the age of 40. Given the context of infection and recurrence of the endometriotic cyst, we decided in our case to carry out radical treatment with right adnexectomy.

Concerning postoperative management, endometriosis requires treatment and continuous monitoring, which does not stop immediately postoperatively. The literature covers publications concerning the postoperative medical treatment of endometriosis and endometriomas with follow-ups, data collection methods, and very variable treatment modalities. A recent randomized study compared six months of immediate postoperative treatment in 259 patients divided into four groups: placebo, LH-RH analogue or continuous estrogen-progestin contraception and diet alone. At 18 months, no significant difference was found in terms of recurrence; only the rate of dysmenorrhea was significantly lower in the group having received treatment with an LH-RH analogue. In our case, the patient benefited from an LH-RH analog injection. Follow-up demonstrated a reduction in dysmenorrhea at the 3rd and 6th months postoperatively.

## 4 Conclusion

Infection of an endometriosic cyst is an exceptional complication. A few rare cases have been described in the literature. Our case was characterized by the occurrence of a serious complication such as pelvic peritonitis. Management was carried out by emergency laparoscopy with right adnexectomy. We emphasize the particularities of this surgery, namely careful dissection to avoid secondary lesions, especially digestive. Finally, we must not forget post-operative care and medical treatment, which must be discussed case by case depending on the clinical signs and the patient's wishes.

## Consent

Written informed consent was obtained from the patient for publication of this case report and accompanying images. A copy of the written consent is available for review by the Editor-in-Chief of this journal on request.

## Ethical approval

Ethical approval is not applicable. This work is not containing any personal information. Faculty of Medicine (Mohammed VI University of Sciences and Health UM6SS. Casablanca. Morocco) confirms that case report or case series do not require ethics approval.

## Funding

This research did not receive any specific grant from funding agencies in the public, commercial, or not for profit sectors.

## Author contribution

All the authors have read and agreed to the final manuscript.

## Guarantor

Abderrahim Siati

## Conflict of interest statement

The authors declare that they have no competing interests.
